# VTA GABA neurons modulate specific learning behaviors through the control of dopamine and cholinergic systems

**DOI:** 10.3389/fnbeh.2014.00008

**Published:** 2014-01-22

**Authors:** Meaghan C. Creed, Niels R. Ntamati, Kelly R. Tan

**Affiliations:** Department of Basic Neurosciences, Medical Faculty, University of GenevaGeneva, Switzerland

**Keywords:** benzodiazepine, dopamine, acetylcholine, NAc, VTA, interneuron, pharmacogenetics, optogenetics

## Abstract

The mesolimbic reward system is primarily comprised of the ventral tegmental area (VTA) and the nucleus accumbens (NAc) as well as their afferent and efferent connections. This circuitry is essential for learning about stimuli associated with motivationally-relevant outcomes. Moreover, addictive drugs affect and remodel this system, which may underlie their addictive properties. In addition to dopamine (DA) neurons, the VTA also contains approximately 30% *γ*-aminobutyric acid (GABA) neurons. The task of signaling both rewarding and aversive events from the VTA to the NAc has mostly been ascribed to DA neurons and the role of GABA neurons has been largely neglected until recently. GABA neurons provide local inhibition of DA neurons and also long-range inhibition of projection regions, including the NAc. Here we review studies using a combination of *in vivo* and *ex vivo* electrophysiology, pharmacogenetic and optogenetic manipulations that have characterized the functional neuroanatomy of inhibitory circuits in the mesolimbic system, and describe how GABA neurons of the VTA regulate reward and aversion-related learning. We also discuss pharmacogenetic manipulation of this system with benzodiazepines (BDZs), a class of addictive drugs, which act directly on GABA_A_ receptors located on GABA neurons of the VTA. The results gathered with each of these approaches suggest that VTA GABA neurons bi-directionally modulate activity of local DA neurons, underlying reward or aversion at the behavioral level. Conversely, long-range GABA projections from the VTA to the NAc selectively target cholinergic interneurons (CINs) to pause their firing and temporarily reduce cholinergic tone in the NAc, which modulates associative learning. Further characterization of inhibitory circuit function within and beyond the VTA is needed in order to fully understand the function of the mesolimbic system under normal and pathological conditions.

## Introduction

**Figure 1:**
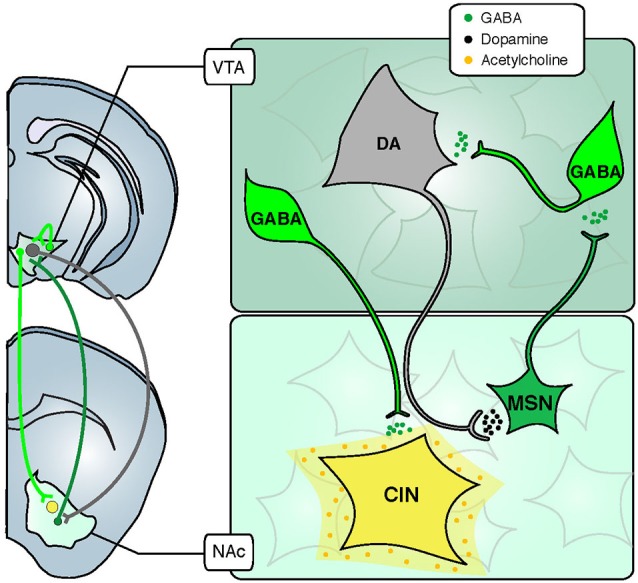
**Inhibitory and modulatory connections between the VTA and the NAc.** The VTA is composed of two major cell types: GABAergic neurons (green) and DA neurons (grey). Glutamate neurons are not schematized for clarity. GABA interneurons control the activity of DA cells that release DA in the NAc through their long-range projections. In turn, MSNs send projections back to the VTA, preferentially inhibiting GABA cells and thus disinhibiting DA neurons. Some GABA neurons also send long-range projections to the NAc, where they selectively inhibit CINs. Abbreviations: VTA: ventral tegmental area; NAc: nucleus accumbens; GABA: *γ*-aminobutyric acid; DA: dopamine; CIN: cholinergic interneuron; MSN: medium sized-spiny neuron.

Learning about motivationally relevant stimuli in the environment is critical for all aspects of survival, from feeding and reproduction to avoiding dangerous or aversive situations. This learning is primarily mediated by the mesolimbic system. Often referred to as the reward system, the mesolimbic system is primarily comprised of the ventral tegmental area (VTA) and the nucleus accumbens (NAc) as well as their afferent and efferent connections (Figure 1). Signaling the salience of an event or stimulus from the VTA in the midbrain to the NAc and other forebrain regions has largely been ascribed to dopamine (DA) neurons (Berridge and Robinson, 1998; Smith et al., 2011). Over the past several decades, extensive studies at the cellular and behavioral levels have shown that populations of DA neurons in the VTA increase their behavior in response to rewarding stimuli (Schultz et al., 1993). In response to aversive stimuli, the activity of most DA neurons is silenced (Mileykovskiy and Morales, 2011; Cohen et al., 2012), although a subset of DA neurons show activation (Joshua et al., 2008). Over training with reward experience, this DA activation will occur not only in response to the salient primary stimuli *per se*, but will also occur in response to previously neutral cues in the environment that the animal has associated with these motivationally relevant outcomes (Schultz, 2013). As a consequence of DA activity, DA in the NAc induces a motivational drive, and this DA signal is modulated by past experience of reward and punishment (Oleson et al., 2012; Howe et al., 2013). In this way, mesolimbic DA has been conceptualized as a teaching signal, coding the magnitude of aversive and rewarding environmental stimuli and increasing behavioral vigor related to these salient stimuli (Peciña and Berridge, 2013). The rewarding effect of addictive drugs is also mediated by mesolimbic DA. Following exposure to drugs of abuse, DA is elevated throughout the mesolimbic system (Lüscher and Ungless, 2006). This persistent increase in DA results in pathological salience attributed to drug-associated cues, and compulsive drug use despite negative consequences. In this way, drug addiction is an example of adverse behavioral consequences arising from a malfunction of the mesolimbic system.

As previously discussed, the role of DA in the mesolimbic system has been the subject of intense investigation over the past several decades. However, in addition to DA, the VTA also contains approximately 30% *γ*-aminobutyric acid (GABA) neurons, and the role of these inhibitory neurons in the mesolimbic system is less well understood. Within the VTA, there are two general populations of GABA neurons: interneurons, which provide local inhibition of DA neurons and projection neurons, which provide long-range inhibition of multiple brain areas including the NAc (Figure 1). In general, inhibition is critical for regulating neuronal excitability, and allows flexibility in circuit connectivity. A consequence of this flexibility is plasticity within the mesolimbic system, which permits reward-related learning. Despite their likely functional consequences, the role of VTA GABA neurons in reward- and aversion-related learning is not well understood. Until recently, it has been difficult to disentangle the function of DA neurons and GABA neurons, and between local VTA GABA neurons and GABAergic projection neurons (GPNs).

In this review we discuss studies using optogenetic and pharmacogenetic tools to dissect the precise role of GABAergic neurons in the mesolimbic system. Much of this recent work has characterized the functional neuroanatomy of inhibitory circuits in the mesolimbic system, and has begun to elucidate the precise mechanisms by which these inhibitory circuits regulate activity of DA neurons and other neuromodulatory systems, such as acetylcholine (ACh). It is now evident that these inhibitory circuits critically regulate DA neuron function and reward-related learning. VTA GABA neurons bi-directionally modulate activity of local DA neurons, which underlies reward or aversion at the behavioral level. Conversely, long-range GABA projections from the VTA to the NAc selectively target cholinergic interneurons (CINs; Figure 1), regulating local ACh release and modulating associative learning. We also use the example of drug addiction to discuss how inhibitory neurons contribute to dysfunction of the mesolimbic system and consequent maladaptive behaviors. Continuing to clarify the role of inhibitory neurons within the VTA and beyond will be necessary to fully understand the function of the mesolimbic system under normal and pathological conditions.

## Activation of local GABA neurons in the ventral tegmental area (VTA)

**Figure 2:**
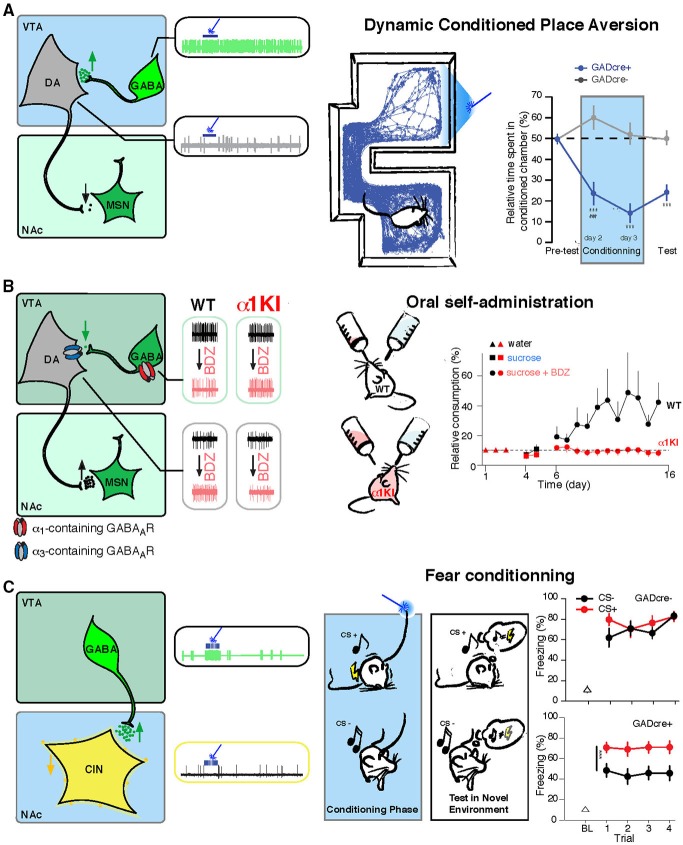
**VTA GABA neurons drive specific behaviors through the control of the DA and cholinergic systems. (A)** Left, VTA GABA neurons are selectively infected to express ChR2-eYFP (green, AAV-ChR2-eYFP-flox in the VTA of GADcre+ mice). Middle, *in vivo* example trace of single unit recording of a GABA and a DA cell showing that blue-light stimulation induces a time locked increase of firing rate in infected GABA neurons and as a consequence a shut down in the activity of DA cells. Right, dynamic conditioned place aversion (CPA): GADcre+ mice developed an aversion for the blue-light paired chamber compared to GADcre− mice. This is shown on the example tracking trace of a GADcre+ mouse and on the aversive learning curve plotting the relative time spent in the conditioned chamber for both groups of mice. **(B)** Left, the ventral tegmental area (VTA) contains DA neurons expressing α3-containing GABA_A_ receptors and GABA cells that express α1-containing GABA_A_ receptors. Middle, *in vivo* example trace of single unit recording of GABA and DA cells before and after midazolam (MDZ) tail vein injection. GABA neurons decrease their basal firing rate, leading to the disinhibiton of DA cells, which increase their firing rate. These effects are abolished in α1-knock-in mice in which GABA_A_ receptors containing the α1 subunit isoform are insensitive to BDZs. Right, oral-self administration of MDZ paradigm: WT mice preferred to consume from the MDZ containing solution whereas this preference is not developed in α1-knock-in mice. **(C)** Left, same preparation as in **B**, VTA GABA projection neurons selectively inhibit CIN of the NAc. Middle, *in vivo* example trace of single unit recording of GABA and CIN cells showing that in response to blue-light stimulation CIN are completely inhibited due to the excitation of VTA GABA cells. Right, activation of these fibers during the association of a conditioned stimulus (CS+, white noise) to an aversive outcome (brief mild footshock) enhances the ability to subsequently discriminate between the conditioned stimulus and the unconditioned stimulus (CS, pure tone) that was not paired with an aversive outcome, as shown on the graph for the percentage of freezing measured on the test day in response to CS+ and CS− for both groups of mice. Abbreviations: VTA: ventral tegmental area; NAc: nucleus accumbens; WT: wild type mouse; α1KI: α1 subunit point mutant knock-in mouse; BDZ: benzodiazepine; GABA: *γ*-aminobutyric acid; DA: dopamine; CIN: cholinergic interneuron; MSN: medium sized-spiny neuron. Adapted from Tan et al. (2010, 2012) and Brown et al. (2012).

As mentioned, the VTA contains a large proportion of GABA neurons (~30%; Dobi et al., 2010). We now appreciate the intimate role these neurons play in regulating activity of DA neurons, and the behavioral consequences of their activity. GABA released from local VTA neurons profoundly affects the activity of VTA DA neurons. Using Cre mice (GAD-cre (Tan et al., 2012) or VGAT-cre (van Zessen et al., 2012) to express channelrhodopsin 2 (ChR2) selectively in GABAergic neurons), it has been possible to selectively manipulate VTA GABA neurons *in vitro* and *in vivo*, and determine the effects on DA neurons as well as the consequences of this activation on behavior (Figure 2). Using *in vivo* extracellular recording in VTA ChR2 injected and anesthetized GAD-cre mice to monitor DA neuron activity, we have shown that driving activity of VTA GABA neurons strongly inhibits DA neuron spontaneous firing rate (Tan et al., 2012; Figure 2A). In stark contrast, shutting down the activity of GABA neurons, by expressing the proton pump halorhodpsin in VTA GABA neurons, leads to an increase or disinhibition of DA cells (Bocklisch et al., 2013). These results were confirmed with *ex vivo* patch clamp electrophysiology, showing that local VTA GABA neurons make direct synaptic connections with DA neurons. Blue-light activation of ChR2-expressing GABA neurons induced inhibitory post-synaptic responses in neighboring DA neurons. These light-evoked currents were abolished by the sodium channel blocker tetrodotoxin or by the chloride channel blocker picrotoxin, confirming a monosynaptic connection mediated by GABA_A_ receptors (Tan et al., 2012; van Zessen et al., 2012). Through activation of GABA_A_ receptors, local GABA cells control their target DA neurons, decreasing their excitability, thereby balancing excitation from glutamatergic inputs (Overton and Clark, 1992).

This inhibitory control has important consequences for DA function and downstream behavior, particularly regarding significance to motivationally relevant outcomes (Schultz et al., 1997; Schultz, 2006; Fields et al., 2007; Bromberg-Martin et al., 2010). Specifically, exposure to salient but aversive stimuli can lead to the inhibition of VTA DA neurons (Mirenowicz and Schultz, 1996; Ungless et al., 2004, 2010; Brischoux et al., 2009; Matsumoto and Hikosaka, 2009; Hong et al., 2011; Zweifel et al., 2011; Tan et al., 2012; van Zessen et al., 2012). Recordings in anesthetized animals have demonstrated that aversive stimuli (such as a foot-shock) transiently and potently increase the spontaneous firing rate of GABAergic neurons of the VTA and decrease the activity of DA neurons (Ungless et al., 2004; Hong et al., 2011; Tan et al., 2012). Likewise, *in vivo* recording in awake behaving animals determined that aversive stimuli lead to excitation of the majority of VTA GABA neurons, which were optogenetically identified (Cohen et al., 2012). Conversely, mixed responses were observed in DA neurons, with the predominant response being inhibition (Brischoux et al., 2009; Cohen et al., 2012; Tan et al., 2012). The increase in GABA neuron spontaneous firing rate and decrease in DA neuron activity following the aversive stimuli of a foot-shock were prevented by a GABA_A_ receptor antagonist, but were not affected by DA antagonists (Tan et al., 2012), indicating that the effects on DA firing rate were downstream of the increased activity of GABA neurons.

Confirming the regulatory role of VTA GABA neurons on motivationally relevant behaviors, driving local VTA GABA neurons with ChR2 results in strong aversion to a compartment associated with light stimulation (Tan et al., 2012). In this dynamic conditioned place aversion (CPA) paradigm (also named real time CPA task), the VTA of ChR2-infected GAD-cre mice was stimulated with blue light when the animal was exploring one of the two compartments, and the light was turned off as soon as the mice exited this chamber. On the test day, where no blue light is applied to the VTA, an aversion of the light paired-chamber was measured (Figure 2A). Interestingly, the CPA produced in response to activating VTA GABA neurons persisted during test sessions, indicating a strong learning effect resulting from DA neuron inhibition. Furthermore, driving VTA GABA neurons also disrupted reward consumption, as measured in a free-sucrose access paradigm (van Zessen et al., 2012). In another study, *in vivo* recordings of the activity of VTA cells were done while mice learned to associate an odor with appetitive rewarding or aversive outcomes. GABA neurons increased their firing during the delay between a conditioned stimulus (odor) and an unconditioned stimulus (reward or aversive air puff), while the firing rate was not changed during the delivery of the reward or punishment. The authors speculated that this ramping up of GABAergic firing may modulate DA activity during reward expectation and may help compute the reward prediction error (Cohen et al., 2012). Such cellular mechanism of action has been correlated with subsecond DA release measurements in the NAc of rat undergoing fear-conditioning tasks. These studies showed that a warning signal presentation (Oleson et al., 2012; Olesson and Cheer, 2013) or a fear conditioning cue (Badrinarayan et al., 2012) result in decreased DA levels as measured by fast scan cyclic voltametry.

Taken together, these observations implicate VTA GABA neuron activity in learning motivationally relevant outcomes associated with unconditioned stimuli. Specifically, driving VTA GABA neurons leads to negative modulation of DA neurons and aversion at the behavioral level. This learning signal may have implications for the involvement of VTA GABA neurons in maladaptive learned behaviors, such as those that characterized in drug addiction.

## Inhibition of local GABA neurons in the ventral tegmental area (VTA)

VTA GABA neurons receive inhibitory inputs mainly from medium sized-spiny neuron (MSN) of the NAc (Xia et al., 2011). Activation of MSNs with ChR2, for instance, leads to the inhibition of VTA GABA cells, and consequent disinhibition of DA neurons (Bocklisch et al., 2013). This mechanism of cellular disinhibition is implicated in the rewarding effects of several classes of addictive drugs (Lüscher and Ungless, 2006). Specifically, opioids (Johnson and North, 1992a), gamma-hydroxy butyric acid (GHB) (Cruz et al., 2004), cannabinoids (Oleson and Cheer, 2012) and benzodiazepines (BDZs; Tan et al., 2010, 2011) act on VTA GABA neurons (Johnson and North, 1992b) and inhibit their activity. We and others have examined how decreasing the activity of VTA GABA neurons, contributes to the synaptic and behavioral adaptations induced by addictive drugs.

BDZs are a class of drug used in clinic to treat anxiety, insomnia and muscle spasms. They also induce sedation and anterograde amnesia and unfortunately, they carry the potential for addiction. All these effects are mediated through BDZs action as positive allosteric modulators of GABA_A_ receptors; they potentiate inhibition in the brain. *In vivo* administration of BDZs inhibits the activity of VTA GABA neurons and consequently increases DA neuron’s firing rate (Figure 2B). Along with BDZs, opioids are another class of addictive drugs that act initially on GABA neurons through binding to μ-opioid receptors specifically expressed on GABA cells (Pickel et al., 2002), decreasing their activity thus leading to the disinhibition of VTA DA neurons (Johnson and North, 1992a).

All addictive drugs, such as cocaine, amphetamine, morphine, GHB, nicotine and ethanol induce characteristic forms of synaptic plasticity on VTA DA neurons within 24 h following an acute exposure (Lüscher and Malenka, 2011). Specifically, an increase in excitatory synaptic strength (as measured by the ratio of the amplitude of AMPA to NMDA-mediated currents (Ungless et al., 2001)) is observed, as well as inward rectification of the AMPA current at positive potentials (Bellone and Lüscher, 2006), which results from the insertion of calcium-permeable AMPA receptors. Interestingly, activation of DA neurons is sufficient to induce this plasticity, since optogenetic activation of these neurons alone (using DA transporter-cre mice) induces the same form of plasticity (Brown et al., 2010). BDZs also trigger this critical feature (Heikkinen et al., 2009; Tan et al., 2010) indicating that they share a common mechanism of DA regulation with other classes of addictive drugs.

Using a pharmacogenetic approach, we demonstrated that the effect of BDZs and more specifically midazolam (MDZ, a BDZ potentiating all GABA_A_ receptors) on neuronal activity and plasticity are mediated by α1-containing GABA_A_ receptors. In the α1(H101R) point mutant mouse line, which carries a single nucleotide mutation that disrupts binding of BDZs to the GABA_A_R binding site (Rudolph et al., 1999), all effects of MDZ are abolished (Tan et al., 2010). Moreover, unlike wildtype mice, these α1(H101R) mice did not self-administer MDZ in an oral-self administration task where they had the option to drink from two bottles, differing only in that one contains a MDZ (Figure 2B).

Inhibition of VTA GABA cells results in the release of the inhibitory break onto DA neurons. Such disinhibition represents a means to trigger the release of DA in target brain regions to modulate synaptic transmission and hence regulate specific behaviors such as the oral-self administration of BDZs, a reward-related behavior.

## Long-range GABAergic projections from the ventral tegmental area (VTA)

VTA DA neurons are long-range projection neurons, which target the prefrontal cortex and the NAc, among other areas. These same areas are also innervated by a subset of VTA GPNs; these neurons comprise approximately 25% of VTA GABA cells (Margolis et al., 2006; Figure 1). In the NAc, GPNs target CINs nearly exclusively, with very sparse innervation of MSNs (Brown et al., 2012). Conversely, VTA DA neurons modulate both MSNs and CINs, either through volume transmission or direct synaptic contacts (Moss and Bolam, 2008).

Using *ex vivo* whole cell recording methods in GAD-cre mice expressing ChR2 in VTA GABA cells, we showed that the connection between VTA GPNs and CINs in the NAc is monosynaptic. Light-evoked currents were recorded in 100% of CINs and blocked by picrotoxin or tetrodotoxin, whereas such currents were rarely detected in MSNs or parvalbumin-expressing interneurons (Brown et al., 2012). *In vivo* recordings confirmed that activating VTA GABA neurons at physiological frequencies potently inhibited CINs for the duration of the stimulation (Figure 2C). This anatomical and functional connectivity has important consequences for associative learning. In a fear conditioning task, when GPNs were optogenetically activated during presentation of a specific tone paired with a foot-shock, mice showed increased ability to discriminate this conditioned tone from an unconditioned tone (Brown et al., 2012; Figure 2C).

During Pavlovian conditioning, VTA DA neurons increase their firing in response to a stimulus predicting either reward or aversion resulting in increased DA release in the NAc. In parallel, the inhibition of CINs produced by GPNs would modulate information processing by MSNs either directly through the actions of muscarinic receptors (Surmeier et al., 2007) or by increasing DA release from VTA terminal fields in the NAc (Cragg and Rice, 2004). Moreover, CINs express DA D2 receptors and are subject to DA regulation (Alcantara et al., 2003) and tonic activity of CINs elicits DA release (Cachope et al., 2012). In this way, through their actions on MSNs and CINs, VTA GABA and DA neurons interact to coordinate activity and reward processing of the NAc. Interestingly, however, inhibiting GPNs terminating in the NAc was insufficient to disrupt reward consumption. These observations support a dual mechanism of VTA to NAc communication, in which targeting of NAc CINs by VTA GPNs and of MSNs and CINs by DA neurons operates in concert to enable the association of motivationally relevant outcomes to unconditioned stimuli.

## Concluding remarks

Within the mesolimbic reward system, communication of rewarding and aversive stimuli has largely been ascribed to DA neurons. The firing rate of DA neurons responds to salient stimuli that are both rewarding and aversive in valence, and is involved in computing reward prediction error. These DA neurons project to regions throughout the brain, including the NAc, which is implicated in behavioral responses to rewarding and aversive stimuli. As such, the mesolimbic DA system is critically involved in learning about motivationally-relevant outcomes. However, in addition to DA cells, the VTA contains GABA neurons, which synapse locally onto DA neurons, and send long-range projections to brain areas innervated by VTA DA neurons. Until recently, the role of VTA GABA neurons on the regulation of DA neurons and on reward-learning was not well understood. However, with the advent of selective optogenetic and pharmacogenetic tools, the role of these inhibitory neurons within the mesolimbic reward system has begun to be elucidated.

We have discussed how within the VTA, aversive stimuli drive the activity of GABA neurons, leading to inhibition of DA neuron firing. Conversely, inhibiting the activity of these VTA GABA neurons (for example, by administration of BDZs) increases the activity of DA neurons and is behaviorally rewarding. The contribution of long-range GABA projection neurons to mesolimbic system function, and the interaction of GPNs with DA projection neurons at the level of their projection areas have also begun to be investigated. For example, despite the established role of the NAc in the maintenance of reward-related behavior (From Introduction), optical inhibition of GPN afferents in the NAc had no effect on reward consumption (van Zessen et al., 2012), and produces no aversive state *per se* (Brown et al., 2012). By confirming that disruption of GABAergic signaling from VTA GPNs to the NAc is insufficient to disrupt reward or produce aversion, these observations further underscore the critical role of DA projections in reward-related learning and coding the valence of environmental stimuli. However, driving activity of GPNs from the VTA to NAc enhances associative learning despite this lack of effect on consumatory behavior or aversion. These observations lead us to the question of the heterogeneity of VTA GABA cells. Within the NAc, antidromic optical activation of VTA GABA neurons inhibited approximately 25% of VTA DA neurons (Brown et al., 2012), suggesting that only a subset of VTA GABA neurons project to the NAc and some others have local collaterals. In addition the population of GABA interneurons that do not project outside the VTA, it is likely that a distinct set of GABA neurons project to other brain regions involved in reward processing and behavior, such as the PFC and hippocampus.

To fully appreciate the role of inhibition in the mesolimbic system, a better understanding of upstream inputs onto VTA GABA neurons, and the role of GABA neurons originating in other nodes of the mesolimbic system are needed. Optogenetic manipulations have recently been used to explore both of these issues. Using these tools to map functional connectivity, it was revealed that both bed-nucleus of the stria terminalis (BNST) glutamatergic and GABAergic projections preferentially innervate putative GABA neurons in the VTA (Jennings et al., 2013).* In vivo* photostimulation of BNST glutamatergic projections resulted in aversive and anxiogenic behavioral phenotypes. Conversely, activation of BNST GABAergic projections produced rewarding and anxiolytic phenotypes, similarly to direct activation of VTA GABA cells (Jennings et al., 2013). Another source of input onto VTA GABA cells is through back-projections from the NAc. Dopamine receptor type 1 (D1R)-receptor-expressing MSNs, but not Dopamine receptor type 2 (D2R)-expressing MSNs of the NAc form functional synaptic contacts with GABA neurons in the VTA (Xia et al., 2011; Bocklisch et al., 2013). This connection is potentiated following exposure to cocaine (Bocklisch et al., 2013), and inhibitory transmission from VTA back to the NAc is suppressed (Ishikawa et al., 2013). These studies provide crucial information about upstream inputs onto VTA GABA neurons and may provide further insight into how DA release is controlled under normal and pathological conditions.

DA modulation is important for function of reward, aversion and reward related learning. While much work has been devoted to studying the function of DA neurons and their role in encoding salient information of rewarding or aversive in nature, the role of VTA GABA neurons has been less well characterized. However, as we have discussed, it is now understood that GABA neurons regulate the activity of both VTA DA neurons and target neurons in VTA projection areas. This intimate, bi-directional modulation of DA neurons not only contributes to reward prediction error and DA plasticity, but also encodes the valence state of rewarding and aversive stimuli and also helps orchestrate behavioral responses to salient stimuli. Further characterization of inhibitory circuit function within and beyond the VTA will be necessary to fully understand the function of the mesolimbic system under normal and pathological conditions.

## Conflict of interest statement

The authors declare that the research was conducted in the absence of any commercial or financial relationships that could be construed as a potential conflict of interest.
